# Health-related quality of life after severe trauma and available PROMS: an updated review (part I)

**DOI:** 10.1007/s00068-022-02178-5

**Published:** 2022-11-29

**Authors:** Annesimone Lotfalla, Jens Halm, Tim Schepers, Georgios Giannakópoulos

**Affiliations:** Department of Trauma Surgery, Trauma Unit, Amsterdam University Medical Center, Location Academic Medical Center, Meibergdreef 9, 1105 AZ Amsterdam, The Netherlands

**Keywords:** Health-related quality of life, Injuries, Instruments, Literature review, Multiple trauma, Patient-outcome assessment, Patient-reported outcomes, Polytrauma, QoL assessment

## Abstract

**Introduction:**

Throughout the years, a decreasing trend in mortality rate has been demonstrated in patients suffering severe trauma. This increases the relevance of documentation of other outcomes for this population, including patient-reported outcome measures (PROMs), such as health-related quality of life (HRQoL). The aim of this review was to summarize the results of the studies that have been conducted regarding HRQoL in severely injured patients (as defined by the articles’ authors). Also, we present the instruments that are used most frequently to assess HRQoL in patients suffering severe trauma.

**Methods:**

A literature search was conducted in the Cochrane Library, EMBASE, PubMed, and Web of Science for articles published from inception until the 1st of January 2022. Reference lists of included articles were reviewed as well. Studies were considered eligible when a population of patients with major, multiple or severe injury and/or polytrauma was included, well-defined by means of an ISS-threshold, and the outcome of interest was described in terms of (HR)QoL. A narrative design was chosen for this review.

**Results:**

The search strategy identified 1583 articles, which were reduced to 113 after application of the eligibility criteria. In total, nineteen instruments were used to assess HRQoL. The SF-36 was used most frequently, followed by the EQ-5D and SF-12. HRQoL in patients with severe trauma was often compared to normative population norms or pre-injury status, and was found to be reduced in both cases, regardless of the tool used to assess this outcome. Some studies demonstrated higher scoring of the patients over time, suggesting improved HRQoL after considerable time after severe trauma.

**Conclusion:**

HRQoL in severely injured patients is overall reduced, regardless of the instrument used to assess it. The instruments that were used most frequently to assess HRQoL were the SF-36 and EQ-5D. Future research is needed to shed light on the consequences of the reduced HRQoL in this population. We recommend routine assessment and documentation of HRQoL in severely injured patients.

## Introduction

Worldwide, traumatic injury is one of the three leading causes of death in patients between the ages of 5 to 44 years [1]. For patients categorized as having severe or profound injuries defined as an Injury Severity Score (ISS) that is equal or above 16, a higher risk of mortality is predicted [2]. The number of patients that do not survive severe trauma, however, is decreasing, due to improvements in surgical procedures, centralization of trauma care and reduction in number of trauma incidents [3–12]. This increases the importance of documentation of outcomes other than mortality of severely injured patients on the short- and long-term, since not much is known about these patients’ way to recovery and the quantification of the impact of the trauma on the whole population (e.g., by means of Disability Adjusted Life Years). Furthermore, since patients have an increased chance of survival, they will have to face the consequences of their trauma for a longer period which might influence their health-related quality of life (HRQoL). In his dissertation, Lansink et al. [13] suggested that monitoring of quality of life should be the focus of research from now on. Based on the relatively low numbers of preventable deaths, further reduction in mortality is after all expected to be small. Hence, what is now increasingly of more relevance, is life *after* severe trauma.

The World Health Organization (WHO) defines quality of life (QoL) as the individuals’ perception of their position in life in the context of the culture and value systems in which they live and in relation to their goals, expectations, standards, and concerns [14]. Although often—incorrectly—interchangeably used in literature, health-related QoL (HRQoL) forms a separate construct, which is defined by the Centres for Disease Control and Prevention (CDC) as an individual’s’ perception on physical and mental health and its impact on QoL [15]. Both definitions clarify that (HR)QoL is an individual, subjective perception, which can only be assessed by numerous questionnaires that are either generic or disease-specific; the former facilitate the possibility to compare different diseases with each other, whereas the latter identify the specific effects of, for example, traumatic injuries and allow comparisons with healthy individuals. HRQoL-measurement is valuable, as it enables caregivers to offer tailor-made aftercare to patients. Not only does this benefit the individual patient, but also the policy makers and the general society, since trauma injuries cause substantial medical and societal costs [16, 17].

The terms *polytrauma, major trauma,* and *severe injury,* which are commonly used interchangeably in literature, refer to the situation in which patients experience multiple injuries, often leading to impairments in physiologic state [18–20]. Although the cut-off value of ≥ 16 is used most often internationally for identifying polytrauma due to its ability of predicting the risk of mortality [26], multiple definitions in terms of ISS values are reported in literature for terms as ‘severe trauma’ or ‘major injury’ [21–25], leaving no consensus regarding ISS thresholds for these populations. Regardless, not only a high risk of mortality (> 10%) [26] is to be expected for those patients, but studies also demonstrate higher morbidity rates in comparison to patients suffering minor trauma [27, 28], leading to the hypothesis that HRQoL is probably significantly impacted.

To our knowledge, there have been only a few reviews of the literature published to summarize evidence regarding HRQoL after severe traumatic injuries [29, 30]. Since patients with an ISS > 15 were excluded in one review [29], studies on injury-specific HRQoL in the other [30], and emphasis was placed on instruments and methods used to measure HRQoL in both [29, 30], we aim in this review to summarize the study results discussing the HRQoL of patients with severe trauma, as defined by an article’s given ISS-threshold. Furthermore, available patients reported outcome measures (PROMs) will be presented.

## Methods

### Search strategy

An exhaustive search was conducted in the Cochrane Library, EMBASE, PubMed, and Web of Science covering the population of patients with severe trauma and the outcome of interest (i.e., HRQoL) defined in the introduction. All articles published from inception to the 1st of January 2022 were included and imported in Rayyan QCRI [31]. In addition to database-searched articles, references of (Cochrane) reviews and included articles and excluded reviews were checked and screened for title and abstract by two reviewers.

The used search strategies that were applied were adjusted to the syntax appropriate for that database and are reported as full texts in Electronic supplementary material (ESM) Appendix A. There were no restrictions with regard to the year of publication.

### Eligibility criteria

Studies that met all of the following criteria were included:

I. Studies have the objective to describe QoL or HRQoL (since both terms are used interchangeably in literature) of patients that, according to their own given definition that is well-described by means of an ISS-threshold value, have suffered major trauma, severe injury or polytrauma (e.g., defined as a population ISS ≥ 16—other thresholds as adopted by authors defining major or severe trauma, however, were accepted as well, since we aimed to cover literature’s HRQoL data on severe trauma patients, taking into account the different ISS thresholds that exist for severely injured patients) *or* have the objective to describe instruments used to assess QoL or HRQoL in patients with severe trauma;
II. The publication is an original article;
III. The article is published in English, German, French, Arabic or Dutch (selection of languages is based on the authors’ language skills);
IV. The full text of the article is available.

Studies on general injury populations and injury-specific studies were both included. Multiple independent populations included in one study were considered separately.

The following articles were excluded:

I. Studies that included exclusively patients described as having suffered mild or minor trauma;
II. Studies using exclusively trauma severity indices scores other than ISS;
III. Studies not defining the patient reported outcome (PRO) explicitly in terms of (HR)QoL (but in less standardized terms such as disability, (dis)function or (dis)satisfaction);
IV. Books, commentaries, letters, reports, (conference) abstracts, posters, presentations, discussion papers, (systematic) reviews, editorials.

### Data extraction

Data-extraction from the included studies was done in duplicate to avoid errors and missing relevant data.

From each eligible study, the following data-elements were extracted into a Microsoft Excel sheet:

- Study characteristics: first author’s last name, year of publication, geographical location, study design, number of participants;
- Population characteristics: age at baseline, gender, median/mean ISS, general population description (e.g., mechanism of injury), duration of ICU and hospital stay;
- PRO characteristics: reported PRO, instrument used to assess PRO, elements observed with instrument, follow-up duration/time point of outcome assessment;
- Outcome: results summarization, author conclusion.

### Synthesis of results

A narrative design was chosen for this review since a meta-analysis could not be produced due to the heterogeneity of the included studies. HRQoL will be discussed per instrument used to assess it in a separate manner.

## Results

### Literature search

**Fig. 1:**
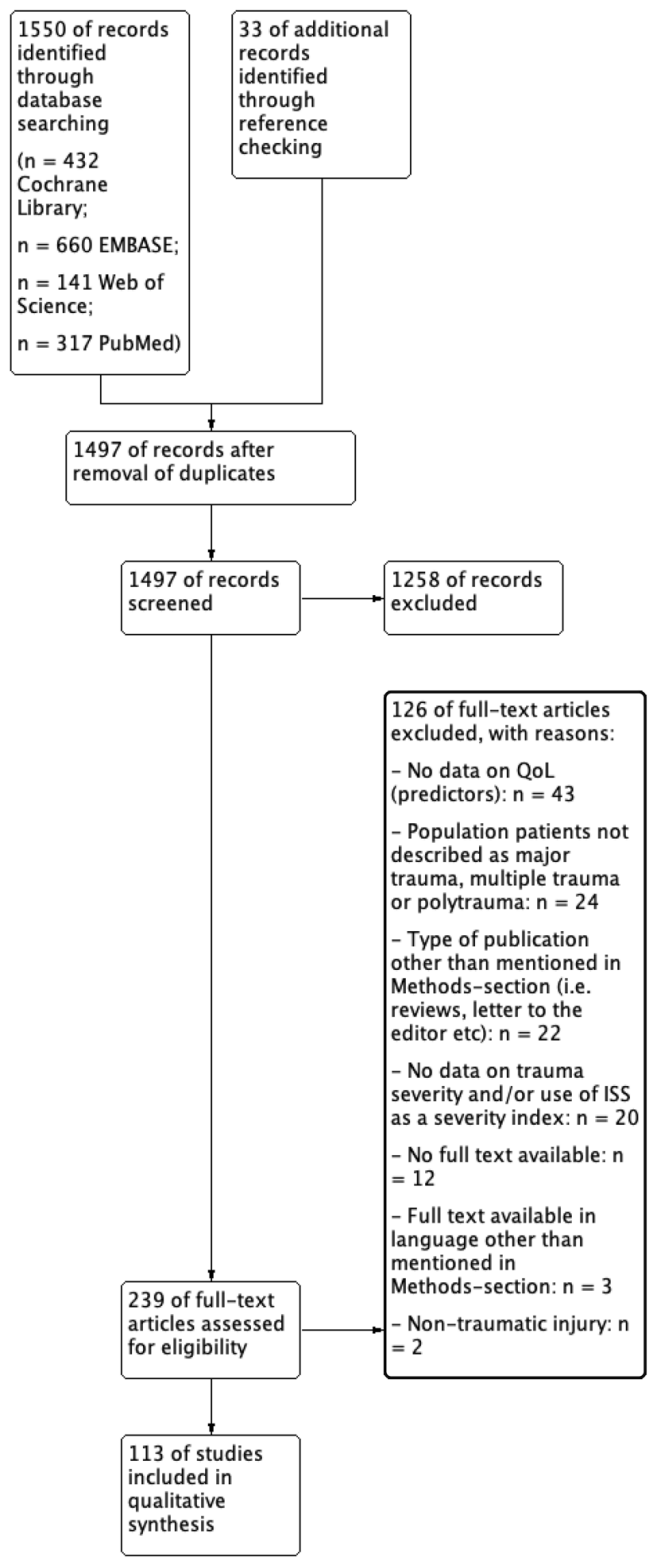
Flowchart of study inclusion during the search and review process

The search strategy identified 1550 articles of potentially relevant articles, while reference checking yielded an additional 33 articles (in total *n* = 1583 articles). Removal of duplicates and screening of titles and abstracts resulted in a selection of 239 articles that appeared to be relevant to the review subject; after applying the eligibility criteria on the full text papers, 113 articles were included for the final analysis. The main reasons for exclusion of publications were lack of data on this review’s desired outcomes, and inclusion of populations that were not necessarily identified as having suffered major trauma (e.g., populations with general trauma). The study flow diagram is shown in Fig. 1.

### General study characteristics

In total, 113 studies were included, all published between January 1994 and January 2022. The general characteristics of the studies are presented in Table 1 (ESM Appendix B). In all studies, the outcomes were defined in terms of HRQoL or QoL. The majority of studies were conducted prospectively (*n* = 65) [32–72, 158, 161–163, 166–169, 171, 173, 176, 177, 181–183, 185, 186, 189–193, 196, 197] and in Germany (*n* = 26) [33, 36, 39, 41, 45, 46, 49, 51, 53, 57, 58, 67, 69, 70, 73, 75–83, 189, 191]. Investigated populations were identified as having suffered major trauma, severe and/or multiple injury or polytrauma, as defined by the eligibility criteria; studies’ average ISS ranged from 9 to 57.

Sample sizes ranged between 8 [84] and 1482 [188], with the majority of patients being male (> 50% in 98 articles [32–52, 54–83, 86–97, 158, 159, 161, 163, 165–168, 170–183, 185–194, 197–199]). Age of the participants in the included studies ranged between 2 [192] and 92 [44, 48] years. Five studies [85, 163, 167, 192, 199] assessed HRQoL in children and/or adolescents (≤ 18 years) only.

### Methods to measure QoL

Nineteen instruments were used to assess HRQoL (an overview of characteristics and descriptions of each instrument is presented in Table 2 (ESM Appendix C)). Of those instruments, the 36-item Short-Form (SF-36) was used most frequently (*n* = 40) [37, 39, 41–44, 47, 50, 52, 61–65, 68, 71–73, 76, 77, 81, 90, 95, 97–99, 160, 161, 165, 168–170, 173, 176, 181–183, 186, 195, 196] followed by the EuroQoL-5 Dimension Questionnaire (EQ-5D, *n* = 39) [38, 40, 44, 47, 53, 55–57, 60, 64, 65, 68, 76, 77, 84, 88, 91, 92, 94, 96, 100, 167, 171, 176–178, 180–182, 185, 186, 190–195, 198, 199] and 12-itemed Short-Form (SF-12) (*n* = 17) [45, 46, 66, 67, 69, 70, 79, 80, 82, 83, 89, 172, 174, 175, 177, 187, 188]. A substantial number of studies (*n* = 32) used multiple tools to measure the HRQoL [38, 44–47, 64–70, 76, 77, 79, 80, 82, 83, 96, 161, 167, 169, 176–178, 181, 182, 186, 192, 195, 196, 199]. The Polytrauma Outcome (POLO) chart, which combines the Trauma Outcome Profile (TOP), Glasgow Outcome Scale (GOS), EQ-5D and SF-36, has been validated recently by multiple studies, and was used in 6 articles [49, 58, 74, 75, 78, 189]. The remaining studies used the TOP (*n* = 11) [47, 48, 51, 65, 68, 76, 77, 93, 179, 181, 182], Hannover Score for Polytrauma Outcome (HASPOC, *n* = 10) [45, 46, 66, 67, 69, 70, 79, 80, 82, 83], a subjective scale (*n* = 5) [33, 36, 38, 59, 61], the Sickness Impact Profile (SIP, *n* = 5) [38, 54, 87, 159, 161], the Quality-of-Wellbeing scale (QWB scale, *n* = 4) [34, 158, 162, 163], the Paediatric Quality of Life Inventory (PedsQL) (*n* = 3) [167, 192, 199], the Nottingham Health Profile (NHP, *n* = 3) [35, 47, 196], the Life satisfaction checklist (LiSat9 and LiSat11, *n* = 2) [86, 169], The Quality of Life after Brain Injury (QOLIBRI, *n* = 2) [65, 181], the Trauma-specific QoL questionnaire (T-QoL) (*n* = 2) [184, 197], the Classification of disability by the International Classification of Impairment, Disabilities and Handicaps (*n* = 1) [164], the Hadorn’s QoL and health questionnaire (*n* = 1) [101], the Quality-of-Life-questionnaire (*n* = 1) [32], the St. George’s Respiratory questionnaire (*n* = 1) [166], and the RAND-36 (*n* = 1) [85]. The latter tool includes the same item-set as the SF-36 but differs in scoring; it will therefore be discussed separately.

### Overall QoL

The results are presented per instrument measuring HRQoL and in order of frequency of usage. In Table 1 (ESM Appendix B) the results are summarized and presented mostly per HRQoL domain or subscale.

#### SF-36

HRQoL as measured by the SF-36 was overall reduced [37, 42, 47, 50, 61–64, 68, 71, 76, 77, 81, 95, 97, 98, 160, 168–170, 173, 176, 183, 186, 196] when compared to pre-injury status or population norms. In some studies SF-36 scores of certain domains (e.g., physical and social functioning) improved significantly over time, although remaining significantly below population norms [37, 39, 42, 50, 52, 61, 63, 71, 161, 183, 186] up to one to fifteen years after the traumatic event [42, 47, 50, 52, 61–64, 68, 71, 77, 81, 97, 161, 183, 186].

Although HRQoL was reduced generally in all polytrauma patients, traumatic brain injury (TBI) impacted HRQoL significantly more when present, in comparison to patients with an ISS ≥ 16 without TBI [39, 47, 61, 62, 65]. Again, time had a beneficial effect on SF-scores of most domains, as shown by an increasing trend, indicating an overall improvement of HRQoL over time (i.e., 12 months [39]).

Severe trauma resulted in early retirement up to 20% of the population [170], and reduced ability to resume employment up to 30% [176, 182, 186, 196]. Moreover, scores in certain SF-36 domains resulted in approximately half of patients losing their ability to return to work after 5 years [71]. Although both patients returning and patients not returning to work report lower HRQoL scores in *some* of the SF-36 domains compared to the general population, patients not returning to work tend to show lower scores for *all* subscales. A higher percentage of TBI patients showed a decrease in their capacity to work when compared to non-TBI patients [47].

Furthermore, lower HRQoL in patients with severe trauma results in reported fatigue, reduced activity, reduced motivation, less frequent participation in and satisfaction with social activities, and the feeling of being more restricted in daily activities [42, 97]. The number of patients with depression and/or anxiety after polytrauma decreased after at least 6 months in patients receiving cognitive behavioural therapy shortly after the event leading to injury [41].

No comparisons were made in some studies [43, 73, 165, 181, 182, 195], hence making it impossible to compare HRQoL between populations, pre-injury status and between time points.

#### EQ-5D

HRQoL of patients suffering severe trauma was below population norms [40, 53, 57, 64, 76, 88, 97, 167, 171, 176, 178, 185, 191, 194, 198] and lower than pre-injury HRQoL [44, 47, 55, 56, 185, 186, 192–194] in most studies. Furthermore, patients with an ISS ≥ 16 reported a lower HRQoL than did patients with an ISS < 16 [53, 55]. The majority of patients reports having problems regarding HRQoL up to several years after the traumatic incident [40, 44, 47, 53, 55–57, 59, 60, 64, 76, 77, 84, 91, 96, 177, 180–182, 190, 195, 199]. Some studies report an improvement in HRQoL-scores over time [53, 57, 176, 177, 185, 192, 193]. Children and teenagers with major trauma reported both similar [167] and reduced [199] HRQoL compared to healthy peers in different studies.

Patients with a flail chest and/or rib fractures show comparable HRQoL scores to control groups with healthy adults [92]. Patients with Lisfranc and/or Chopart injuries report significantly lower HRQoL compared to the general (non-trauma) population, but similar HRQoL to general trauma patients [178].

Patients with a femoral fracture with a pelvic or acetabular fracture (i.e., floating hip) reported reduced HRQoL, although no significant difference was found when compared to patients with isolated acetabular or femoral fractures. However, depending on the location of the fracture in floating hip patients, significant differences in HRQoL can be detected within a minimum of 7 years post-trauma [100].

#### SF-12 and HASPOC

Ten studies [45, 46, 66, 67, 69, 70, 79, 80, 82, 83] used both the SF-12 and HASPOC as a measurement of QoL.

Polytrauma patients presented with reduced HRQoL when compared to normative values [46]. No difference in HRQoL between TBI and non-TBI patients was found [46, 80]; spinal injuries, however, had deleterious effects on HRQoL, especially in paraplegic patients [69]. Also, mentally impaired patients presented with worse HRQoL after severe trauma in comparison to mentally healthy patients [79].

Although no difference in HRQoL between athletes and non-athletes could be demonstrated, the impairments were serious and of long-term influence [66].

Patients with and without upper extremity trauma reported comparable HRQoL [67, 80], but involvement of the brachial plexus in the polytrauma resulted in significantly lower scores [67]. Worse HRQoL-scores were present in patients with lower extremity injury [69], especially when present below the knee joint [83].

In studies that used SF-12 exclusively, comparable HRQoL-scores in polytrauma patients and the general population were found in one study [89], although impairment in some domains were present in others [172, 174, 177]; significant HRQoL-differences between TBI and non-TBI patients, however, could be demonstrated after more than ten years follow-up, especially in mental and psychological domains [89]. In spine polytrauma patients significantly higher satisfaction on mental domains were found than on physical domains [174]. PCS- and MCS-scores improved, although little, up to 24 months after injury [172, 177], although in populations with multiple fractured ribs [175, 188] and midfoot and/or hindfoot fractures [187] population norms could not be achieved [175].

#### POLO and TOP

Most studies [47–49, 51, 58, 74–78, 93, 179, 181, 182, 189] demonstrated reduced HRQoL in severely injured patients several months post-injury (up to several years), defined as reduced scores in at least one of the domains.

#### Subjective scale

In patients with a suicidal origin of polytrauma, overall outcome was good in half of the patients according to the subjective scale by means of which HRQoL was measured, despite the double adverse influence of severe multiple blunt trauma and psychiatric disorder [33, 36]. 

Patients surviving trauma resulting in an ISS ≥ 25 had significantly lower HRQoL [54]. A study including military service members experiencing polytrauma revealed reduced QoL as well [59].

#### Sickness impact profile (SIP)

Most adult patients ≤ 65 years presented with good QoL after severe trauma; the majority showed no disablement according to the SIP. Approximately one tenth of the patients showed severe disablement (defined as a SIP ≥ 20). Two years after the injury, however, only a slight difference in QoL was found when compared to the general population. Also, SIP-scores did not differ significantly between patients with no, mild-moderate and severe TBI [87]. In another study [54], with inclusion of patients > 65 years as well, however, most patients (63%) reported severe disability (SIP ≥ 20), which was defined as a low QoL. SIP work scores remained significantly higher compared to baseline up to 12 months post-trauma, indicating worse employment status in patients suffering major trauma [161].

Patients sustaining pelvic ring fractures reported mild disability after one year [159].

#### Quality of well-being (QWB) scale

While healthy adults usually score between 0.830 and 0.900 on the QWB-scale, patients with an ISS ≥ 16 scored 0.400 on average. HRQoL improved after 3, 6, 12, 18 and 24 months but remained low [34, 158, 162]; even in adolescent populations, QWB-scores remained below scores of uninjured adolescents [163].

#### PedsQL

Psychosocial health, although improving over time, was reduced in children one-year post-trauma, whereas baseline physical health scores were achieved eventually [192, 199]. Severely injured children showed comparable mean score on the PedsQL to population norm values 6 to 8 years post-trauma [167].

#### Nottingham health profile (NHP)

Most patients showed problems in at least one QoL-domain, as well as disability in undertaking daily activities up to 5 years post-trauma [35, 196]. Almost half of the patients was unable to work afterwards [35]. In a population with severe trauma, TBI patients presented with lower HRQoL than non-TBI patients [47].

#### Life satisfaction checklist (LiSat 9/11)

Three years after severe trauma several domains of HRQoL according to the LiSat9 were affected; 87% of the patients reported lower life satisfaction in at least one domain [86].

After two years, patients with surgically treated pelvic fractured reported poorer HRQoL according to all eleven items of the LiSat 11 compared to a matched normative population, despite good radiologic results [169].

#### QOLIBRI

Although total QOLIBRI score is similar in both major and non-major TBI inflicted polytrauma patients, polytrauma patients with major TBI are cognitively more impaired than do patients with mild or no TBI. In total, approximately one fifth of the patients met the study’s definition of impaired HRQoL [65]. Elderly (≥ 80 years) with TBI presented with the poorest HRQoL in one of the studies [181].

#### Trauma-specific QoL questionnaire (T-QoL)

Impairments in everyday daily living activities were found in approximately one third of patients with severe trauma; almost half did not return to work and was diagnosed with PTSD [197]. Up to 12 months post-injury approximately half of the patients reported daily pain [184, 197].

#### The classification of disability by the international classification of impairment, disabilities and handicaps

QoL was reduced as a result of disability in approximately half of the patients sustaining multiple injuries. Significant improvement occurred over time [164].

#### Hadorn's QoL and health questionnaire

Bicycle trauma patients with an ISS ≥ 16 experienced significantly more impairments in several QoL-domains [101] (see Table 1, ESM Appendix B).

#### Quality of life questionnaire score

Quality of life as measured by the QoL questionnaire indicated that polytrauma patients show a significant reduction of capacity for physical effort, which affects work and other activities that are appropriate to age (see Table 1, ESM Appendix B). Half of the patients had normal QoL after 2 years, which suggests an improvement when compared to QoL one year post-polytrauma [32].

#### St. George’s respiratory questionnaire

Patients admitted with multiple traumas including chest trauma presented with reduced HRQoL, possibly related to pulmonary function limitation [166].

#### RAND-36

Nine years post-trauma, paediatric patients (≤ 15 years) surviving polytrauma had no different HRQoL than the healthy reference population [85].

## Discussion

This review aimed to provide a comprehensive insight into the health-related quality of life of patients surviving severe trauma, including the tools used to assess this outcome.

### Summary of main results

In this review, 113 articles were included that evaluated the health-related quality of life in patients with severe trauma. HRQoL in populations with adult patients sustaining severe trauma (e.g., defined as an ISS ≥ 16) is generally reduced when compared to the general population or pre-injury HRQoL, regardless of the tool used to assess this outcome. Interestingly, only paediatric patients (< 16 years) seem to have a similar HRQoL when compared to normative population values [85, 167, 192]. In a substantial number of studies [32, 34, 37, 39, 42, 50, 52, 53, 57, 61, 63, 71, 87, 158, 161, 162, 164, 172, 176, 177, 183, 185, 186, 192, 193, 199] scores in the used HRQoL-instruments increased over time in some domains, suggesting improvement in HRQoL after severe trauma in the months or years to follow after the event leading to the injuries; HRQoL, however, remained generally reduced when compared to population norms or pre-injury status.

The most commonly applied instruments were the SF-36 and EQ-5D; both are so-called generic instruments, suitable for assessing HRQoL in a broad range of patient populations. Of these instruments, the validity is well recognized [102–107], leading to extensive use in trauma populations for baseline and follow up assessment of HRQoL; available country specific population norms for all subscales make meaningful comparisons possible. The TOP, QOLIBRI, HASPOC, T-QoL and St. George’s Respiratory Questionnaire were the only disease-specific instruments that were used in the included articles, the first four assessing specifically the impact of trauma on HRQoL. Although these have not been extensively validated until recently, comparisons to the more validated SF-36 and EQ-5D show comparable and hence promising results [48, 65, 70, 75, 108–112].

### Strengths and weaknesses of the review

There are certain limitations adherent to this review that need to be addressed.

First, no assessment of methodological quality of the included studies was made. Information on the risk of bias in the included studies is therefore lacking. Some studies, however, are expected to be of lesser methodological quality, for example due to the small sample size (e.g., one study [84] had included only eight patients), retrospective nature of analysis [64, 73–92, 94–101, 159, 160, 164, 165, 172, 174, 175, 178, 180, 184, 187, 188, 194, 195, 198, 199], but especially the lack of a control group or comparison with pre-injury QoL. The latter studies, however, were useful for identification of possible predictors and instruments to assess HRQoL.

Second, all studies that were included in this review, presented results of populations that were identified as having sustained either major, severe, multiple injuries and/or polytrauma, hence allowing patients with varying ISS-cut-off values to be included—the average ISS of populations included in this review ranged from 9 to 57. Therefore, we cannot conclude that this review did include polytrauma patients (often defined as an ISS ≥ 16 [27]) exclusively. Of patients with lower trauma severity scores one can expect better HRQoL, although some studies [37, 43, 54, 64, 77, 79, 87, 98, 170] did not find a significant correlation between injury severity and HRQoL. This suggests that the ISS is primarily useful to predict the life-threatening potential of the injury, which is in accordance with the initial design of ISS, which did not include prediction of HRQoL after trauma [113]. Interestingly, Brasel et al. [43] has indeed demonstrated how objective ISS differed significantly from *perceived* injury severity and how patients often overestimate the severity of their injuries, possibly as a consequence of the PTSD they suffered post-injury. Nevertheless, a substantial number of studies [32, 34, 35, 37, 41, 45, 53, 55–58, 60, 61, 68, 81, 101, 163–165, 171, 173, 174, 176, 177, 185, 189, 192, 194, 195, 199] demonstrated how polytrauma patients do have significantly worse QoL than do patients with an ISS below 16, and how increasing ISS correlates with reduced HRQoL, which is in line with our expectations. Evidence regarding this issue is therefore rather conflicting. The aim of this review, however, was to summarize the HRQoL of patients with severe trauma or major injury, terms that are ill-defined but nevertheless used frequently and interchangeably in literature with varying definitions; in our review, ISS-thresholds differed between articles describing the same population, and most commonly ISS cut-offs of 9, 12 and 16 were used to identify these patients. The fact that the results of patients presenting with an ISS less than 16 were included in this review, is therefore not considered to be necessarily a major methodological limitation, since there’s no consensus (yet) regarding a strict threshold for severe trauma or major injury in literature. Moreover, several studies suggest that the use of an ISS ≥ 16 as a threshold excludes relevant data relating to morbidity and QoL and suggest that lower cut-off values should be adopted to allow for evaluation of other outcomes than survival [20, 114, 115]. The ISS-threshold is after all chosen arbitrarily and is primarily correlated with mortality [116–120], and studies have demonstrated that an ISS ≥ 12 functions similarly to the ISS ≥ 16 cut-off [205, 206].

Third, we chose to only include studies using the Injury Severity Score (ISS) as an indicator of trauma severity and hence as the only tool to identify severely injured patients by. It is possible that studies examining HRQoL using other severity scoring systems [e.g., the Revised Trauma Score (RTS) or Abbreviated Injury Scale (AIS)] were excluded, hence making this review not exhaustive regarding included articles.

Despite these limitations, this review can be considered to be the most extensive to date, with inclusion of 113 unique publications, the majority of which being conducted prospectively. Furthermore, this review has included publications from 1994 to 2022, providing sufficient information on the health-related quality of life patients suffering severe trauma throughout the years. Finally, this is the first systematic review to be conducted aimed at patients with severe trauma in specific (to our knowledge), that provides information on their HRQoL as well as on instruments that may assist in assessing this outcome in this specific population.

### Implications for practice and research

The results of this review, which encompasses studies published more than two decades ago and studies published more recently, are sending the alarming message that the health-related quality of life in patients with severe trauma is significantly reduced in comparison to healthy control groups and pre-injury status. This implies that despite the improvements and advances that have been made in the field of trauma surgery, which have benefited outcomes such as survival and mortality, these did not have its beneficial effects on the HRQoL of severely injured patients. Therefore, future research is urgently needed on at least the following topics:

I. The POLO-chart, which includes the trauma-specific Trauma Outcome Profile (TOP) as an instrument to measure HRQoL, has been validated and used in some, but obviously a minority of studies, as is presented in Table 1 (ESM Appendix B); therefore, further research is needed to explore the reasons and motivations of researchers behind selection of HRQoL-instruments. Variation in selection of instruments across studies decreases the potential to compare studies with each other; therefore, we agree with Polinder et al. [30] recommending the use of the same instrument in studies, which encompasses all dimensions relevant for trauma patients and is easy to use, both for surgeons and patients.
II. Further research is needed on the HRQoL of children surviving severe trauma. In this review, only five studies [85, 163, 167, 192, 199] investigated this outcome in paediatric and adolescent patients exclusively. Recently, Martin-Herz [121] has published a review on this topic, presenting that HRQoL was reduced in four out of six studies; however, studies included in the review were limited (*n* = 16), and average ISS in the studies included was below 16 in the majority (*n* = 14) of studies. Therefore, research on HRQoL in severely injured paediatric patients, including suitable, child-friendly instruments needs to receive more attention in the research field.
III. Future research should focus more on the *consequences* of reduced QoL in patients suffering severe trauma. In this review, we have demonstrated how those patients may be suffering more often from psychiatric comorbidities including depression, anxiety, and PTSD, and how they show reduced capacity to work. This list, however, is not exhaustive; therefore, more focus needs to be placed on the possibly detrimental effects of reduced HRQoL on the patient himself/herself and, not irrelevant, also the consequences for his/her direct environment and general society (e.g., in terms of economic problems).
IV. More information is needed on the parameters that influence HRQoL the most. This might facilitate personalized patient care and prediction of long-term outcomes for incoming patients with severe trauma, which might possibly benefit the patients.
V. Future studies should explore the methods of delivery of the various discussed tools assessing HRQoL, as well as discuss their feasibility in clinical practice.

## Conclusions

Health-related quality of life in patients with severe trauma is overall reduced. Instruments used most frequently to assess HRQoL were the SF-36 and EQ-5D. Assessment and documentation of HRQoL in patients with severe trauma should happen on a routine basis in daily clinical practice to make follow-up of those patients possible, which enables detection of changes in terms of improvement or deterioration.
